# Tracking the scaling of urban open spaces in China from 1990 to 2020

**DOI:** 10.1038/s41598-024-62880-9

**Published:** 2024-05-24

**Authors:** Zhibang Xu, Xiaoqi Duan, Ting Lan, Yashi Wu, Cuiping Wang, Yang Zhong, Haowei Wang

**Affiliations:** 1 Key Laboratory of Urban Environment and Health, Institute of Urban Environment, Chinese Academy of Sciences, Xiamen, 361021 China; 2 College of Computer Science and Technology, Guizhou University, Guiyang, 550025 China; 3 College of Harbour and Coastal Engineering, Jimei University, Xiamen, 361021 China; 4 School of Geographic Sciences, Hunan Normal University, Changsha, 410081 China

**Keywords:** Urban scaling laws, Complex urban system, Sustainable development, Urban open space, China, Urban ecology, Environmental economics, Sustainability

## Abstract

Urban open spaces (UOS) are crucial for urban life, offering benefits across individual and societal levels. However, the understanding of the systematic dynamic of UOS scaling with city size and its potential non-linear performance remains a limited clarity area. This study bridges this gap by integrating urban scaling laws with remote sensing data from 1990 to 2020, creating a framework to analyze UOS trends in China. Our findings reveal that UOS growth is sub-linear scaling with city size, exhibiting economies of scale with scaling exponents between 0.55 and 0.65 and suggesting potential shortages. The distribution structure of UOS across cities is becoming increasingly balanced, as indicated by the rising Zipf’s slope from 0.66 to 0.88. Southeastern coastal cities outperform, highlighting spatial variations and path dependency in UOS development. Additionally, using metrics of Scale-adjusted metropolitan indicator (SAMI) and the ratio of open space consumption to population growth rates (OCRPGR), we observe a trend towards more coordinated development between UOS and population, with a declining proportion of uncoordinated cities. Our long-term, large sample coverage study of UOS in China may offer positive significance for urban ecological planning and management in similar rapidly urbanizing countries, contributing to critical insights for quantifying and monitoring urban sustainable development.

## Introduction

Cities have emerged as epicenters of human activity, housing 56% of the global population^1^. Urban open space (UOS) can be understood as any land without buildings captured within city, such as parks, green spaces, or other undeveloped land^2^. Its concept can be traced back to the “Metropolitan Open Space Act” and “Open Space Act” during the late 18^th^ and early nineteenth centuries. UOS provide multiple values such as natural resource protection, history, entertainment, which is an important spatial component for human urban daily life and can provide many physical, environmental, and socioeconomic benefits^3^. Comfortable UOS are not only beneficial to human health^4^, but also attract people to go out for activities^5–7^, thus improving urban vitality. In addition, they are also beneficial to enhancing resident safety and urban resilience in response to various disasters^8^. Therefore, urban open space is one of the critical places for sustainable cities and human settlements and has received widespread attention from urban planning, management, and urban scientific research.

For UOS studies, different scientific communities have different focuses. The urban management community focuses on public organization and relationships related to open spaces, which are often discussed under the rubric of public space. For example, many studies discussed the beneficial interpersonal connections^9^ and sociopolitical development that neighborhoods and diverse cultural groups^10^ gain from open space. There are also studies discussing the management and challenges that urban development brings to open space^11^. From the Garden City movement^12^ to more recent urban sustainability topics^13,14^, equitable access to open spaces has been increasingly discussed as an environmental justice issue. In contrast, Urban planning and architecture communities pay more attention to the form and function of urban open spaces. Within these studies, the majority concentrate on urban green spaces and parks. For example, many studies explore the principles and methods^15^, practices^16^ and sustainability evaluation^17^ of urban green spaces and parks. A considerable number of works concern the social and environmental effects of urban green space and parks, such as on urban renewal^18^, ecosystem services^19^ and microclimate^20^. From the dynamic perspective, Stanley^21^ have sorted out the types of UOS and reviewed cases from different periods to emphasize the diversity and importance of UOS. Spatial models such as Geographical Field models^22^ and Cellular Automata^23^ are subsequently used for the evaluation and simulation of urban open spaces. Overall, most of the above-mentioned studies concern individual cities or local areas within them and are mostly case studies.

Although the study of UOS contains rich interdisciplinary topics and involves local cases at micro and fine scales. However, there is still a lack of full investigation on the dynamics of UOS from an entire perspective for a system of cities. Notably, complexity science is considered a better tool for understanding urban systems^24^. Urban complexity is not only reflected in a single city itself as a complex system but also in the macro-urban complex system composed of cities. Characteristics that individuals do not show often emerge, and even simple laws appear^25^. The urban scaling laws^26^ is a representative one. For example, several studies have identified the scaling characteristics between the area^27^, economic output^28^ and population of major cities and intra-urban^29^ in China. Urban scaling theory has also been used to assess urban land use efficiency^30^, Sustainable Development Goals^31^, venture capital^32^, urbanization process^33^, urban carbon emissions^34^ and other fields. As an important urban component, UOS has multiple natural, economic, social, and ecological benefits for city. Meanwhile, its dynamics is closely related to urban growth and planning. However, whether similar scaling characteristics also exist in UOS and whether it can be applied in UOS assessment has not attracted enough attention. Do UOS exhibit non-linear scaling with city size? What are its spatiotemporal dynamics? How to understand and assess the performance of urban UOS evolution in the context of sustainable development and Urban Scaling? Generally, the knowledge and understanding of UOS at the urban system level is necessary and important, but remain unclear and uninvestigated.

In order to address the research gap mentioned above, our work takes China, one of the world’s largest urban systems, as a case study to comprehensively quantify the UOS dynamics in as many city samples from 1990 to 2020. We applied the urban scaling methodology to combine the dynamics of population, built-up area and UOS. By adopting specific methods such as the Scale-adjusted metropolitan indicators and the ratio of open space consumption to population growth rates (OCRPGR), we aim to deeply understand and evaluate the dynamic patterns and performance of China’s UOS through a systematic lens. Our work may provide potential insights for urban science, urban planning, and management, and help better achieve sustainable urban development.

## Materials and methods

### Data and Study area

As for data sources, we utilized remote sensing datasets and geographic information datasets. Specifically, they are: (1) Remotely sensed impervious surface data, produced by Gong et al^35^, with a spatial resolution of 30 m; (2) Remote sensing water body data, from EU-JRC^36^, also with a spatial resolution of 30 m; (3) Population grid dataset, downloaded from the Resource and Environment Science and Data Center of the Chinese Academy of Sciences, with a spatial resolution of 1 km.

The impervious surface and water body datasets mentioned above are used to identify urban boundaries, urban open spaces, and urban built-up areas. The population grid dataset is used to count urban populations, which are considered to reduce the effects of Modifiable Areal Unit Problem. For specific calculations, the urban boundary is used as the spatial benchmark, and the total population, total open space area, and total built-up area within the urban boundary are counted by GIS overlay.

In terms of the study area, we covered as many cities in mainland China as possible with an area of more than 5 km^2^ and a population of more than 10,000, excluding those with missing data in some years. The number of cities that meet the above conditions at each time point is shown in Table 1. It should be noted that we adopted the more internationally comparable concept of urban agglomeration^37^ rather than the prefecture-level cities in this work. The former defines a city as agglomeration of contiguous built-up areas and the open spaces in and around them^38^. The latter is considered to include rural areas and lacks international comparability^39^.

**Table 1:** The rank-size fitting evolution for urban open space in China from 1990 to 2020.

Year	Zipf’s slope	α of power law fitting	*P*	City counts
1990	0.66	2.52 ± 0.11	0.08	379
1995	0.63	2.58 ± 0.13	0.49	566
2000	0.65	2.54 ± 0.12	0.37	736
2005	0.91	2.10 ± 0.04	0.00	889
2010	0.89	2.12 ± 0.04	0.13	1087
2015	0.81	2.23 ± 0.06	0.68	1246
2020	0.88	2.14 ± 0.05	0.26	1424

*P* values greater than 0.1 indicate goodness of fit.

### Identification of urban open spaces

As mentioned above, the definition of UOS can be traced back to the late eighteenth century. Although the delimitation of UOS in actual planning or construction are not completely consistent among countries, their sharing commonality refers to captured-land without buildings within city^2^. In this work, we use a relatively updated definition of city and UOS, which was developed by Angel et al^38^. Their definition is considered as one of the two recommended by the United Nations^40^. Specifically, UOS encompass captured open spaces (that are fully surrounded by urban built-up area) and fringe open spaces (within a certain range of urban fringe). This includes all types of land cover except for artificial impervious surfaces and water bodies^38^.

For the specific identification of UOS, we used an advanced mapping method called HUBM^41^, which utilizes the cloud computing platform of Google Earth Engine. Specifically, we first identified urban boundary and urban built-up area based on remotely sensed artificial impervious surface and water body through the bottom-up way, and then extract the non-built-up land surrounded by built-up areas or within 1 km of the urban fringe, that is, UOS. This method has been proven to have high accuracy, details can be found in the corresponding references^41^.

### Urban scaling and rank-size laws

We used the widely discussed urban complexity analysis methodology to help understand the long-term dynamics of UOS from the macro systematic level. Urban scaling law is a deconstruction theory of urban complex systems proposed by Bettencourt et al.^26,42^ since 2007. It refers to the quantified relationship between various urban indicators and city size (measured by urban population) in the system of cities conforms to a power function, as follows,

1 $$Y(t)={Y}_{0}N{(t)}^{\beta }$$

where *Y(t)* represents the measure of urban indicator at time *t*, *Y*_*0*_ is a standardized constant, *N(t)* represents the urban population to represent the size of the city, *β* is the scaling exponents. Notably, urban indicators were classified into three urban scaling regimes^26^ that were distinguished according to the quantitative relationship between *β* and 1, ①Sub-linear, *β* ≈ 0.8 < 1, refers to urban indicator related to urban infrastructure, such as built-up are, etc. ② Linear, *β* ≈ 1, is mostly urban indicators related to individual needs, such as the number of job positions, etc. ③ Super-linear, *β* ≈ 1.1–1.3 > 1, refers to indicators related to urban socioeconomic activities, such as the GDP, etc. Based on this theory, we expect that UOS may belong to a sub-linear regime.

The rank-size rules originated in the early twentieth century, with the power-law distribution of population rank in urban systems first observed by Auerbach^43^ and Lotka^44^. At present, it generally refers to the rank distribution of urban indicators in the system of cities subject to the following formula,

2 $${V}_{i}={V}_{1}{R}_{i}^{-q}$$

where *V*_i_ and *R*_i_ are the value and its rank of the urban indicator for city *i*, *V*_1_ refers to the largest value of the urban indicator in the entire urban system. *q* is often referred to as the Zipf’s slope. We use the latest and more robust method^45^ to address the rank-size fitting.

### Scale-adjusted metropolitan indicators

Since the urban scaling law reveals a widespread non-linear scaling relationship between urban indicators and city size, this challenges the applicability of traditional per capita indicators in measuring true local performance. Because the underlying assumption for comparing per capita indicators between cities is the linear growth of indicators to population. In this regard, the *Scale-adjusted metropolitan indicators* (SAMI) was proposed^46^ to help eliminate the scale effect to discover and quantify the local performance. The calculation formula of SAMI is as follows,

3 $${\xi }_{i}=log\frac{{Y}_{i}}{Y({N}_{i})}=log\frac{{Y}_{i}}{{Y}_{0}{N}_{i}^{\beta }}$$

where ${\xi }_{i}$ is the SAMI for city *i*, *Y*_i_ refers to the actual metric of urban indicator and *Y(N*_i_*)* refer to the expected indicator value according to the urban scaling law, which expresses the average behavior of urban indicator for a city of population *N*_i_.

### New indicator for serving SDG 11

The United Nations' Sustainable Development Goals include SDG11, aimed at urban areas. Specifically, Target 11.3 seeks to enhance inclusive and sustainable urbanization by 2030. To measure progress towards this target, Indicator 11.3.1, the Land Consumption Rate to Population Growth Rate (LCRPGR), was introduced, gauging the ratio of urban land use expansion to population growth. However, LCRPGR alone may not fully encompass the sustainability aspects of urban sprawl, particularly regarding ecological sustainability. Meanwhile, Indicator 11.7.1 focuses on the “Average share of the built-up area of cities that is open space for public use for all, by sex, age, and persons with disabilities”, highlighting the importance of accessible urban open spaces (UOS) in sustainable urban development. To bridge this gap and better assess urban ecological sustainability, we propose a novel metric, the Open space Consumption Rate to Population Growth Rate (OCRPGR). This metric aims to evaluate the harmony between UOS development and population dynamics, offering a more comprehensive tool for understanding urbanization’s ecological sustainability aspects. OCRPGR is defined as follows:

4 $$\text{OCRPGR}=\frac{OCR}{PGR}=log\frac{LN\left(\frac{{Uos}_{t+n}}{{Uos}_{t}}\right)}{LN\left(\frac{{Pop}_{t+n}}{{Pop}_{t}}\right)}$$

where *UOS*_t+n_ and *UOS*_t_ are the total area of UOS at the final year *t* + *n*, and at the initial year *t*, respectively. *OCR* is short for urban Open space Consumption rate. Population Growth rate (*PGR*) is the natural logarithm of the ratio of urban population at two respective times *t* + *n* and *t*, which is the same as that in the LCRPGR indicator. Generally, OCRPGR measures the degree of coordination between urban open space and urban population changes. If the two change directions are different, that is, one increases and the other decreases, the value of OCRPGR is negative, which means that the development of the two is uncoordinated. If OCRPGR > 0, it means that the two are developing in the same direction. Different coordination degree intervals can be divided according to the value of OCRPGR. When 0 < OCRPGR < 1, it means that the two are highly coordinated, and 1 < OCRPGR < 2 means that the two are low coordinated (UOS changes more than population). Furthermore, 2 < OCRPGR < 4 means UOS changes are at least double but lower than quadruple the one of population, and OCRPGR > 4 means UOS changes are at least quadruple the one of population. Overall, OCRPGR provides a quantitative characterization of the coordination between open space and population dynamics during urbanization.

## Results

### Rank-size evolution of urban open space

Based on the area of UOS, we quantified the evolution of the rank-size distribution of UOS for China’s urban system from 1990 to 2020 (Table 1). The results suggest that the UOS development of the entire system has evolved from being dominated by small and medium-sized cities to being balanced-dominated by cities with different sizes. Specifically, while the number and area of Chinese cities continue to grow, UOS’s Zipf slope shows a fluctuating upward trend. From 0.66 in 1990 to 0.88 in 2020, the ranking of UOS areas tends to be a power law distribution. Although we observed poor fitness in 1990 and 2005, the overall evolutionary trend is clear.

### Urban scaling evolution of urban open space

**Figure 1:**
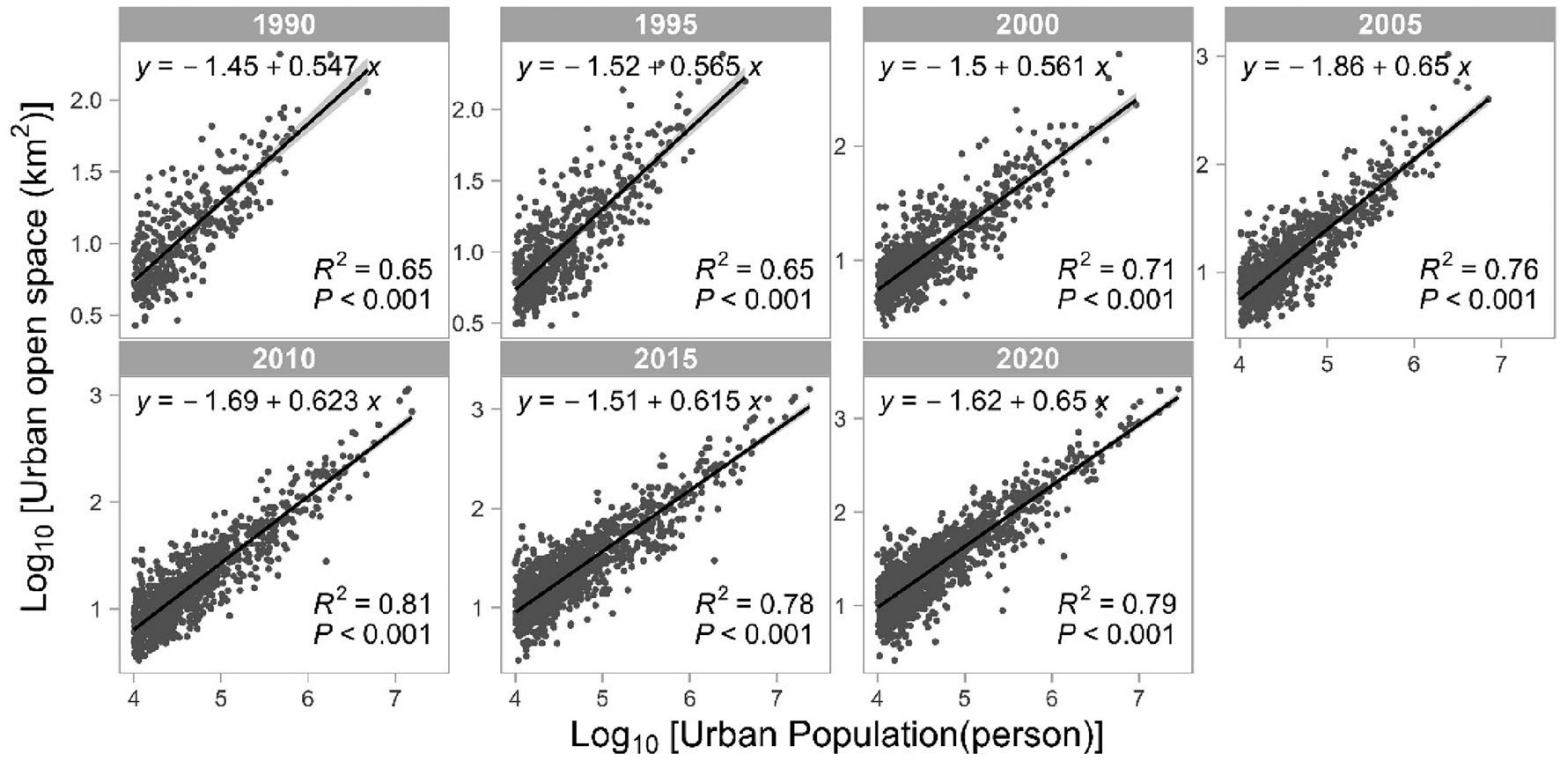
The evolution of Urban Scaling fitting for urban open space and city size in China from 1990 to 2020. Each dot represents a city and each bold black line represents the regression line of urban scaling.

We further performed urban scaling fitting for UOS and city size (quantified by urban population) on every 5-year time slice (Fig. 1). The results show that UOS exhibits a stable sub-linear scaling regime with city size at all time points analyzed. The scaling exponents rose from 0.55 to 0.65 in a tiny move. In other words, UOS generally exhibits economies of scale. The larger the city, the fewer UOS per capita will be. However, judging from the evolution of the 30 years from 1990 to 2020, this decrease process is weakening, which may be related to the inefficient utilization of land that has existed during China’s rapid urban expansion in the past 30 years. Despite this, the scaling exponent of UOS remains at a low level, far lower than the theoretical estimate of 0.8 for urban infrastructure in the hypothesis of urban scaling theory.

### SAMI performance of urban open space for urban complex systems

**Figure 2:**
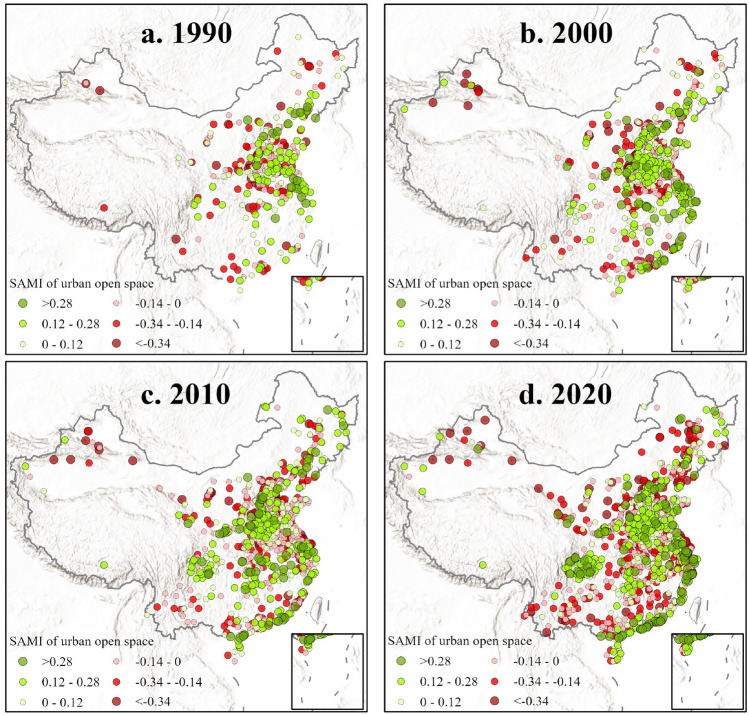
Quantitative local performance of UOS for Chinese cities in each decade from 1990 to 2020 after eliminating scale effects. The green color represents that the UOS performance is better than expected, while the red color represents that it is worse than expected, and the values represents the degree of deviation from expectations.

Since UOS has been in a robust sub-linear scaling regime for 30 years, we use the SAMI equation to eliminate the impact of city size to objectively understand the quantitative local performance of UOS in different cities of the entire urban system. We conducted calculations based on a 10-year time slice (Fig. 2). The green color in the figure represents that the quantitative UOS is better than expected, which suggest UOS has better achieved economies of scale. while the red color represents that it is worse than expected, and the symbol size of the point represents the degree of deviation from expectations. Overall, UOS performance exhibits significant spatial heterogeneity. From a distribution perspective, UOS performance in cities located in the central and eastern China and the Sichuan Basin is generally better, especially in the southeastern coastal areas. From an evolutionary point of view, the performance of UOS shows a certain path dependence, which means its direction of advantages or disadvantages relative to expectations usually does not change in a short period of time. As for key areas, we found that cities in the Yangtze River Delta have shown better UOS performance in the long term, and cities along the coast of Guangdong have always had better UOS performance since 2000. In addition, we also observe that cities with worse-than-expected and better-than-expected UOS both have increased over the past 30 years, and their respective distribution areas have changed little.

### OCRPGR performance of urban open space for human-land relationship

**Figure 3:**
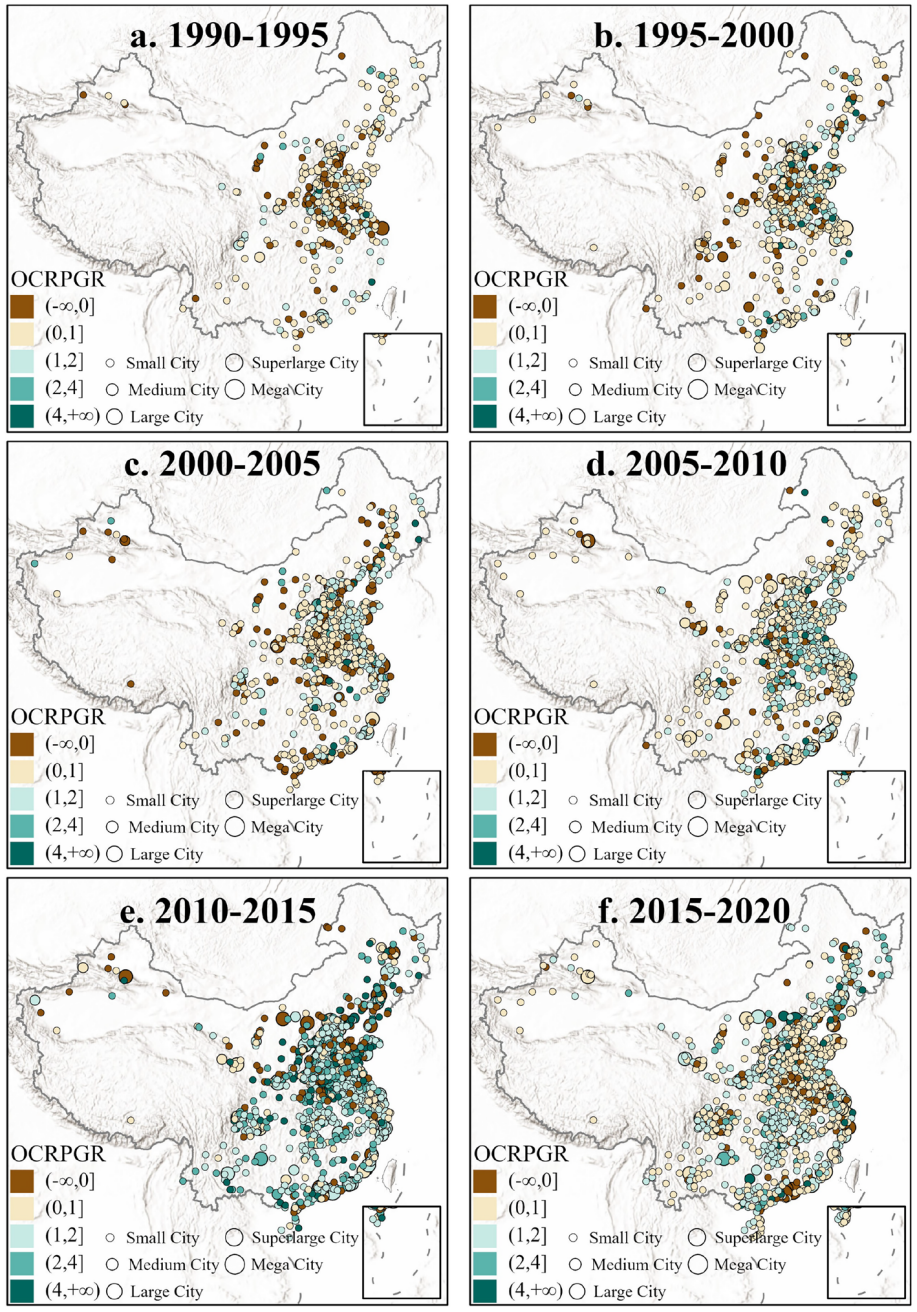
Quantitative OCRPGR for Chinese cities from 1990 to 2020. Each dot represents a city. The OCRPGR is stand for the ratio of Open space Consumption Rate to Population Growth Rate. The distinction of city size comes from the “City Size Classification Standards” published by the State Council of China. Among them, small city refers to cities with a population of less than 500,000, medium city refers to those with a population of more than 500,000 and less than 1 million, large city refers to population between 1 and 5 million, super large city refers to those with more than 5 million and less than 10 million population, mega city refers to urban population of more than 10 million.

The OCRPGR indicator depicts the dynamic relationship between UOS area and urban population size. While reflecting the evolution degree of coordination between them. It can support the assessment of sub-goal 11.3 and 11.7 in the SDG agenda. We quantified OCRPGR in as many cities in China from 1990 to 2020 using time slices separated by 5 years, and identified the spatial distribution dynamics of OCRPGR (Fig. 3). Overall, the spatial differences and agglomeration patterns of OCRPGR are relatively significant, reflecting the complex current situation and significant spatial characteristics of UOS and population change. From a distribution perspective, cities with coordinated UOS and population development are more widely distributed, while cities with uncoordinated (OCRPGR < 0) UOS and population are relatively evenly dispersed in major megalopolis in China. From a dynamic perspective, the proportion of cities where UOS and population evolve in the same direction is increasing year by year, and the distribution of cities where UOS and population are uncoordinated tends to dissipate. In terms of key areas, most cities near Beijing and Shanghai are highly coordinated (0 < OCRPGR < 1), while there are relatively more uncoordinated (OCRPGR < 0) cities in the Greater Bay Area. In some periods, such as 2010 to 2015, the OCRPGR of Chinese cities experienced a more dramatic change than in other periods, and their quantified values were generally higher, which hints at the intensified growth of UOS and possible urban sprawl during this period. After 2015–2020, this phenomenon produced a clear return, seemingly returning to the previous development trend track from 1990 to 2010.

**Figure 4:**
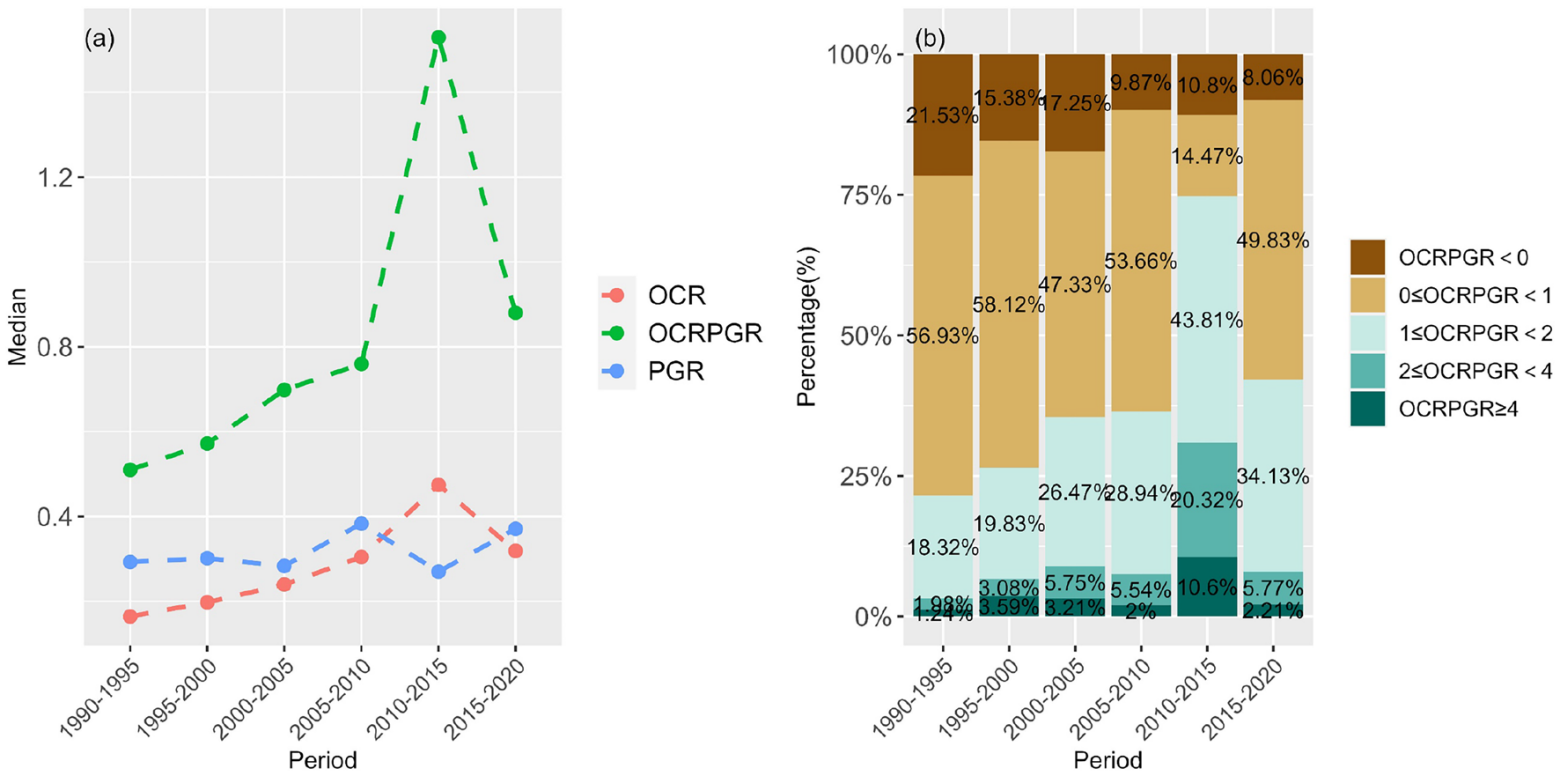
Temporal changes in quantitative values and structural composition of OCRPGR. (**a**) Temporal changes in the median of OCRPGR and its subcomponents OCR and PGR. (**b**) Temporal changes in OCRPGR values in different value intervals. Notes, OCRPGR < 0 means that the changes in urban open space area and population are uncoordinated (the directions of increase and decrease are different), 0 < OCRPGR < 1 means that changes in urban open space area and population are highly coordinated, 1 < OCRPGR < 2 means that the changes in urban open space area and population are low coordinated (urban open space changes more than population), 2 < OCRPGR < 4 means urban open space changes are at least double but lower than quadruple the one of population, OCRPGR > 4 means urban open space changes are at least quadruple the one of population.

Separate quantitative analyses further characterized the temporal evolution characteristics of UOS area and population counts respectively (Fig. 4a). As for UOS, it has maintained a growth trend, with the median of OCR increasing steadily (it fluctuated during 2010–2015 and returned in the next 5 years). The growth in population is relatively more stable, and in most time periods, the median of PGR is higher than OCR. The only time period below OCR (2010–2015) directly led to a sharp increase in the median of OCRPGR. The proportion chart (Fig. 4b) more clearly shows the significant reduction of cities with uncoordinated patterns (OCRPGR < 0) of UOS and population in the entire urban system. However, we also observe that the number of high coordination (0 < OCRPGR < 1) cities also declined compared with the initial time period (1990–1995). This implies that the evolution of UOS and population may have moved from one direction of the balance to the other, a phenomenon that should be paid more attention to in the future.

### The relationship between urban built-up and open space growth

**Figure 5:**
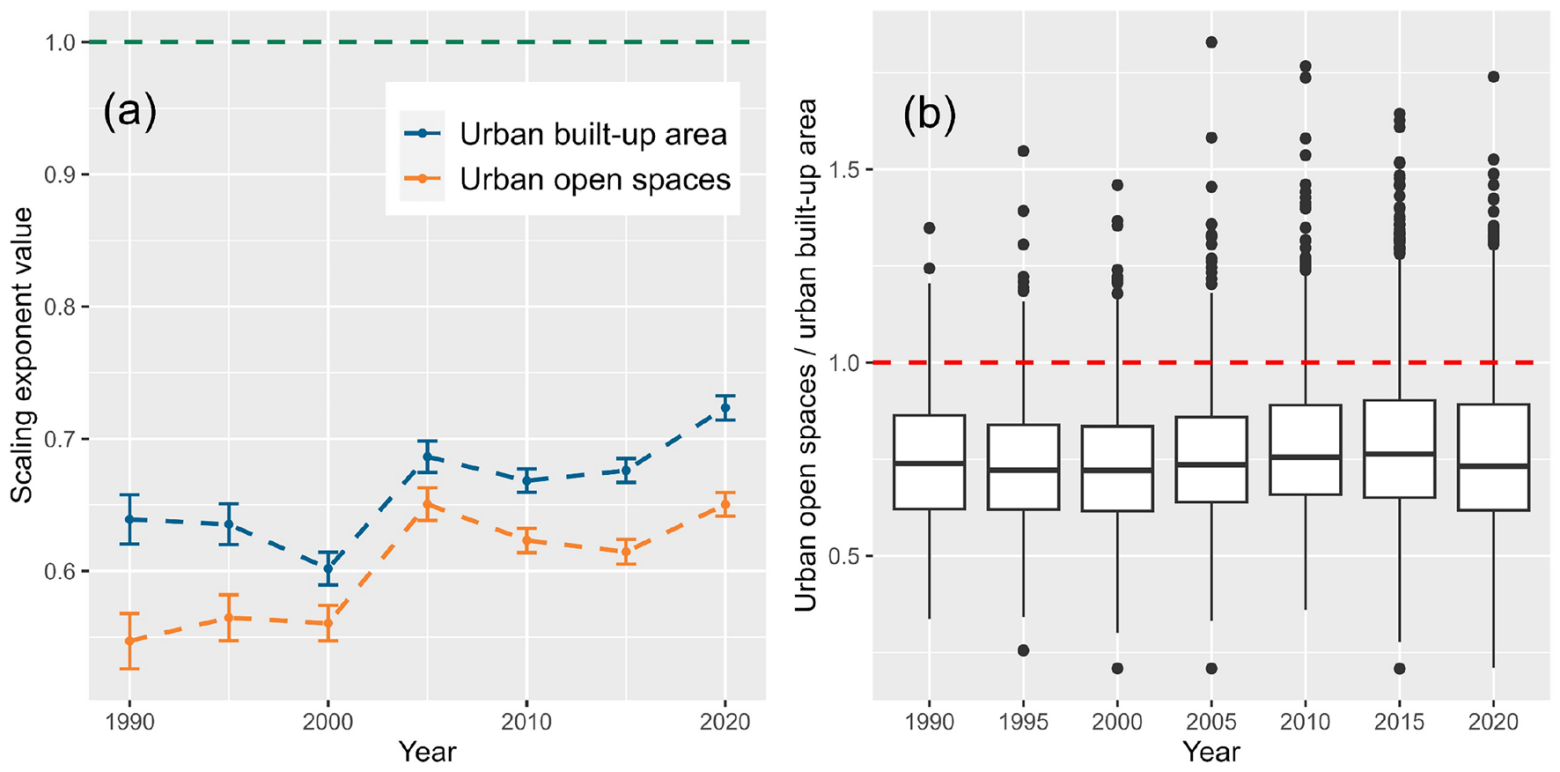
Scaling exponent evolution and ratio distribution for urban open space and urban built-up area. Error bars represent 95% confidence intervals for scaling exponents.

The growth relationship between UOS and urban built-up area (UBA) is also very important. We quantified the dynamics of the scaling exponents of China’s UBA and UOS from 1990 to 2020 (Fig. 5a), and used the area ratio of UOS and UBA as a proxy to analyze the relationship between open space and the built-up area in the system of cities. We found that the dynamic trends of scaling exponents for UOS and UBA are highly similar, which is also closely related to the concept of UOS itself. However, in the context of similar trends, the value gap between the two scaling exponents at different time points is not constant. For example, the quantity difference between the two scaling exponents was small from 2000 to 2005, which implies a small gap in size distribution between UOS and UBA. On the other hand, Fig. 5b shows the stability of the proportion of UOS and UBA, which is reflected in the small fluctuation in the median value of UOS and UBA in the entire system of cities. At the same time, we found that urban outlier samples with a high proportion of UOS tend to increase in number, which indicates that the degree of fragmentation of built-up areas in some cities is too high.

## Discussion

Unlike previous case studies that focused on shorter time periods or focused only on major cities^27^, our work quantifies the dynamics of UOS for all city samples in China during the past 30 years. More notably, we conducted a systematic assessment from an urban system perspective on a consistent spatiotemporal baseline. The spatiotemporal consistent remote sensing quantitates we used are more efficient and cost-effective than previous scaling studies based on statistical data^33^. Basically, our work suggests that the UOS in Chinese cities grows sub-linearly with the growth of city size (measured by urban population), demonstrating the economies of scale in UOS. Our work appears to confirm that UOS utilization may be more efficient in large cities, while implying that excessive open space investment in small and medium-sized cities may not be the optimal-performance choice, either. However, this also depends on the specific economic level and characteristic positioning of the city. In general, our work has advantages over similar studies in terms of time coverage and sample coverage. At the same time, our conclusions also provide further empirical support for the complex system theory of urban scaling laws.

The significant of UOS in urban nature, economy, society, and ecology has been proven in past research^47^. The investigation for scaling of UOS is notable. As UOS refer to non-built lands enclosed or captured by urbanized areas during the urbanization. It can be understood as both a passive outcome and a active choice within the urbanization process. The passive aspect is evident as UOS reflects the transformation and fragmentation of natural land caused by urban activity, thus becoming one of the few natural areas within urban space, although this “naturalness” is relative. On the other hand, the preservation and planning of UOS are integral parts of urban planning, a fact that is evident from the concept’s origin^2^. Furthermore, the passive and active dynamics of UOS in urbanization also significantly affect various practical issues and ideas in urban development, such as “garden cities”^12^ and “compact cities”. The involvement of UOS in urban functionalities^48^ is also considered to affect current hot topics such as carbon emissions^49^ and human wellbeing^50^. Thus, investigating the scaling relationship between UOS and city size is crucial in providing scientific support to address these issues. In this regard, we confirmed the economies of scale of UOS in China and the general inadequacy of UOS supply in China, as indicated by its scaling exponent being lower than theoretical expectations.

The potential policy implications of this work for urban planning and management include, First, SAMI assessment of UOS can provide clear directions for adjusting the total area of open space for cities with different sizes, that is, answer the question of whether it should be increased or decreased. In this process, we should also realize that UOS is not only related to various beneficial effects for humans but also needs to pay attention to the urban sprawl problem that may be exacerbated by excessive development of UOS. Therefore, the reasonable size of UOS is closely related to the urban population. Secondly, our OCRPGR quantification uses a relatively simple metric to further enrich the measure means of UOS assessment, which provides positive insights for other international agendas like SDGs that require cross-national assessments but face difficulties in data availability. This will help to better support the construction of urban ecological sustainability in countries in the Global South. Finally, Thompson^47^ argue the importance of recognizing the value of wild space, informal, loose-fit and sometimes messy spaces. The open space analyzed in this work corresponds to Thompson’s loose definition, which includes all previous surfaces within the city into the category of UOS. Our results also show that the jointly overall open space in China, including parks, green spaces, and these loose areas, still shows a certain degree of undersupply in urban system. In the future, possible directions for our work include further exploring the evolutionary relationship between different populations and culture groups and UOS to help better solve the problem of environmental inequality.

## Conclusion

Urban complexity is reflected in the fact that an urban system composed of cities will emerge with overall characteristics that are not found in a single city. It is from this perspective that this work uses systematic analysis methodologies such as urban scaling laws, combined with modified SDG indicators, to quantify and identify the spatiotemporal characteristics of urban open space evolution in China’s urban system from 1990 to 2020. We found that (1) The evolution of open space in Chinese cities follows a sub-linear scaling regime, with scaling exponents ranging from 0.55 to 0.65. This indicates that the area of UOS exhibits economies of scale as city size increases. However, it also suggests a potential shortage of UOS in urban systems, as the scaling exponents are lower than theoretical expectations. Additionally, the distribution structure of UOS within China’s urban system is increasingly becoming balanced across cities of various sizes. This shift is evident in the rising Zipf’s slope, which has increased from 0.66 to 0.88”. (2) After eliminating the impact of scale benefits, the UOS performance of Chinese cities shows obvious spatial heterogeneity and path dependence, with more cities on the southeastern coast having UOS performance that is better than expected. (3) The OCRPGR indicator results show that China’s UOS and population are developing towards a more coordinated evolution, and the proportion of uncoordinated cities is continuously decreasing. However, open space in some cities is growing faster than population, which may lead to urban sprawl that needs to be treated with caution in the future. (4) Urban open space and the growth of built-up areas are closely related. The median ratio of open space to the built-up area in Chinese cities has been stable for a long time over the past 30 years. Meanwhile, the number of outlier samples with a high proportion of UOS has increased, implying that the degree of fragmentation of built-up areas in some cities may be too high. In general, this work’s long-term, full-sample coverage study of the spatiotemporal evolution of UOS in China can help deepen the systematic understanding of the urban environment and can have positive significance for urban planning and management in rapidly urbanizing developing countries.

## Data Availability

The population distribution data of China that support this study are available for a fee from the Resource and Environment Science and Data Center, Chinese Academy of Sciences (https://www.resdc.cn/DOI/DOI.aspx?DOIID=32). The urban open space datasets generated during this study are available from the corresponding author upon reasonable request.
